# BPD, Not BPD, or Iatrogenic BPD: Findings of Lung Ultrasound Examinations

**DOI:** 10.1097/MD.0000000000000133

**Published:** 2014-11-14

**Authors:** Jing Liu, Shui-Wen Chen, Fang Liu, Yan Wang, Xiang-Yong Kong, Qiu-Ping Li, Jun-Jin Huang

**Affiliations:** From the Department of Neonatology and NICU of Bayi Children's Hospital (JL, S-WC, FL, YW, X-YK, Q-PL, J-JH), Beijing Military General Hospital, Beijing; and Graduate School of Southern Medical University (S-WC, FL, YW), Guangzhou, China.

## Abstract

Lung ultrasound has been extensively used to diagnose many types of lung disease. This study aimed to evaluate the pulmonary reasons for long-term oxygen dependence (LTOD) in premature infants using lung ultrasound.

Lung ultrasound was routinely performed in 50 premature infants clinically diagnosed with bronchopulmonary dysplasia (BPD).

Among the 50 patients studied, there were 9 cases of atelectasis, 4 cases of pneumonia, 2 cases of severe pulmonary edema, and 3 cases of pulmonary edema and consolidation that coexisted with BPD. The oxygen dependence of the babies either completely resolved or significantly decreased following appropriate treatments.

More than one-third of the cases of LTOD in premature babies were caused by either BPD alone or diseases other than BPD. Lung ultrasound plays an important role in differentiating pulmonary causes of LTOD in patients with BPD, and the results of our study suggest that modifying the diagnostic criteria for BPD may be necessary.

## INTRODUCTION

In preterm infants, long-term oxygen dependence (LTOD) is one of the most commonly encountered clinical respiratory problems, particularly in patients with a gestational age (GA) <32 weeks. This condition is often diagnosed as either neonatal chronic lung disease or bronchopulmonary dysplasia (BPD) in cases involving oxygen-dependent infants >28 days of life.^1^ Moreover, lower GA and birth weight are associated with a higher incidence of oxygen dependence. With the improved survival of preterm infants, the incidence of BPD has increased; this finding has serious implications for the survival and quality of life of premature infants affected by this disease. Therefore, the diagnosis and treatment of BPD has become one of the most challenging problems encountered by neonatologists.

We recently conducted lung ultrasound examinations on 50 patients with BPD using the traditional diagnostic criteria.^1^ The results of these assessments showed that a large number of these infants did not actually have BPD, whereas other infants did not have BPD alone (meaning that BPD coexisted with other lung diseases). The LTOD of these infants may have been caused by other disease processes; therefore, it would be unreasonable to diagnose these patients with BPD.

## PATIENTS AND METHODS

### Patients

The Institutional Review Board of the Beijing Military General Hospital, Beijing, China, approved the study protocol (number 2011-LC- Ped-01). From May 2012 to February 2014, 50 newborns diagnosed with BPD were admitted to the Department of Neonatology and the neonatal intensive care unit (NICU) of Bayi Children's Hospital, which is affiliated with the Beijing Military General Hospital. These newborns were subsequently enrolled in our study.

### Lung Ultrasound

Bedside lung ultrasound was performed using a high-frequency linear 9 to 12 MHz probe (GE Voluson E6 or E8; GE Medical Systems, Milwaukee, USA), which was positioned perpendicular to the ribs. The infants were positioned in the supine, lateral, or prone position. Findings from 3 areas of each lung, areas defined by the anterior and posterior axillary lines, were recorded. Ultrasound examination findings, including atelectasis, pneumonia, pulmonary edema, and consolidation, were carefully recorded. Ultrasound diagnoses of atelectasis, pneumonia, or pulmonary edema were made using criteria previously reported in the literature.^2–7^

## RESULTS

**FIGURE 1:**
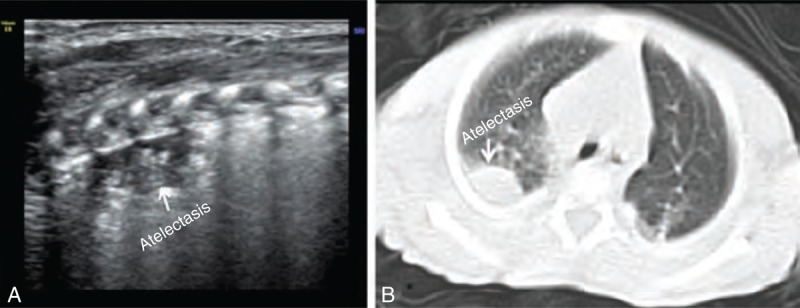
Pulmonary occult atelectasis. GA 26^+2^ weeks, birth weight 900 g, hospitalized because of RDS. This patient required a ventilator for 58 days and oxygen supplementation for 100 days and was diagnosed with BPD clinically. A lung ultrasound showed (A) consolidation and an air bronchogram within 2 intercostal ranges, (B) findings that were confirmed as atelectasis by chest CT scan. This occult atelectasis was the reason for this baby's LTOD. BPD = bronchopulmonary dysplasia, GA = gestational age, LTOD = long-term oxygen dependence, RDS = respiratory distress syndrome.

**FIGURE 2:**
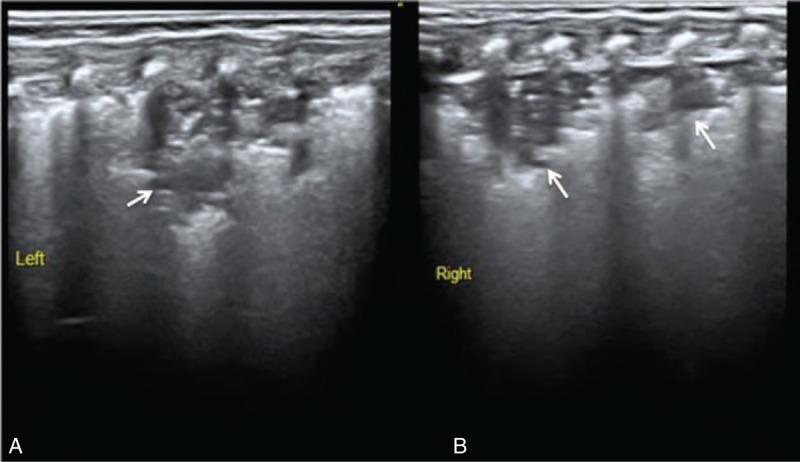
Pneumonia. GA 29^+2^ weeks, birth weight 1200 g, Apgar score 8-10-10 points/1-5-10 min. This patient was maintained on a ventilator because of RDS and was diagnosed with BPD clinically because oxygen therapy was required after 58 days of life. A lung ultrasound showed large consolidations with irregular edges, as well as air bronchograms bilaterally (A—left lung, B—right lung) (arrows). The pleural line was either blurred or disappeared, and the A-line disappeared. This infant was diagnosed with bilateral pneumonia, which was the reason for oxygen dependence. BPD = bronchopulmonary dysplasia, GA = gestational age, RDS = respiratory distress syndrome.

**FIGURE 3:**
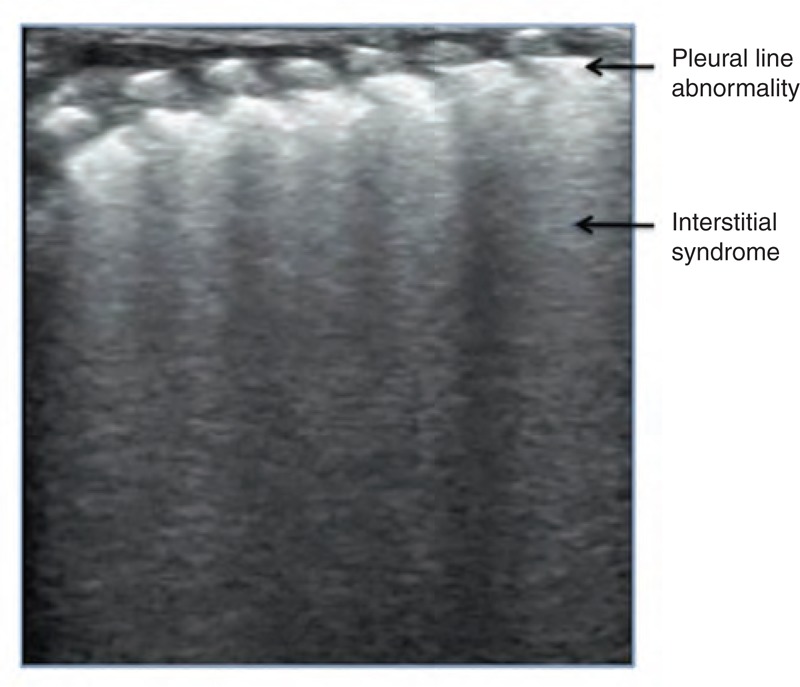
Lung edema. GA 27^+3^ weeks, birth weight 1000 g. This baby presented without spontaneous breathing after birth and was placed on a ventilator because of dyspnea following resuscitation. The patient was diagnosed with BPD clinically because oxygen therapy was required after 40 days of life. A lung ultrasound demonstrated a “white lung” appearance, as well as the coarse appearance of the pleural line in the right lung, suggesting the presence of severe pulmonary edema, which was the reason for this baby's LTOD. BPD = bronchopulmonary dysplasia, GA = gestational age, LTOD = long-term oxygen dependence.

**FIGURE 4:**
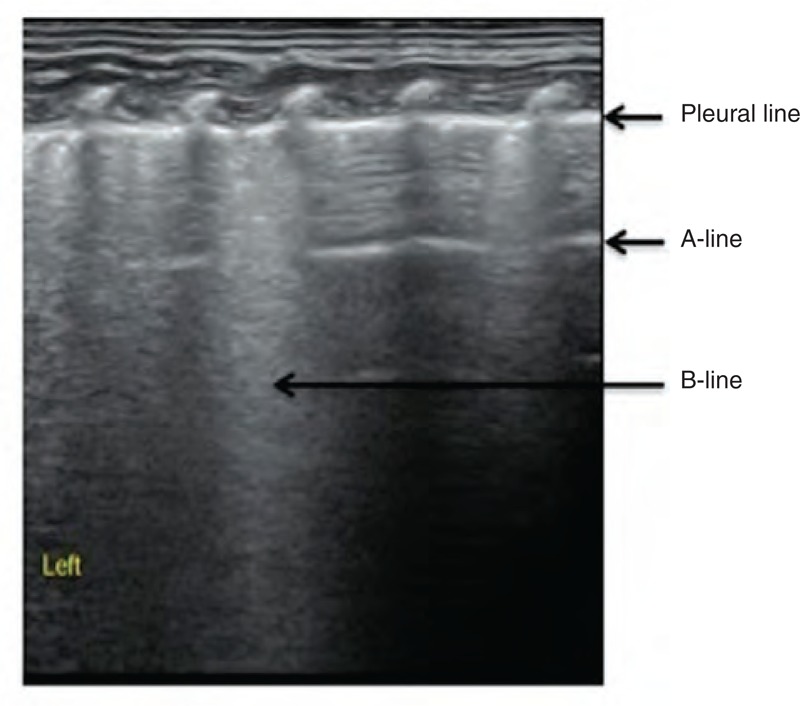
Interstitial syndrome. A lung ultrasound demonstrated interstitial syndrome in the upper field of the left lung, whereas the lower field of this lung was almost normal, suggesting the presence of mild lung edema.

**FIGURE 5:**
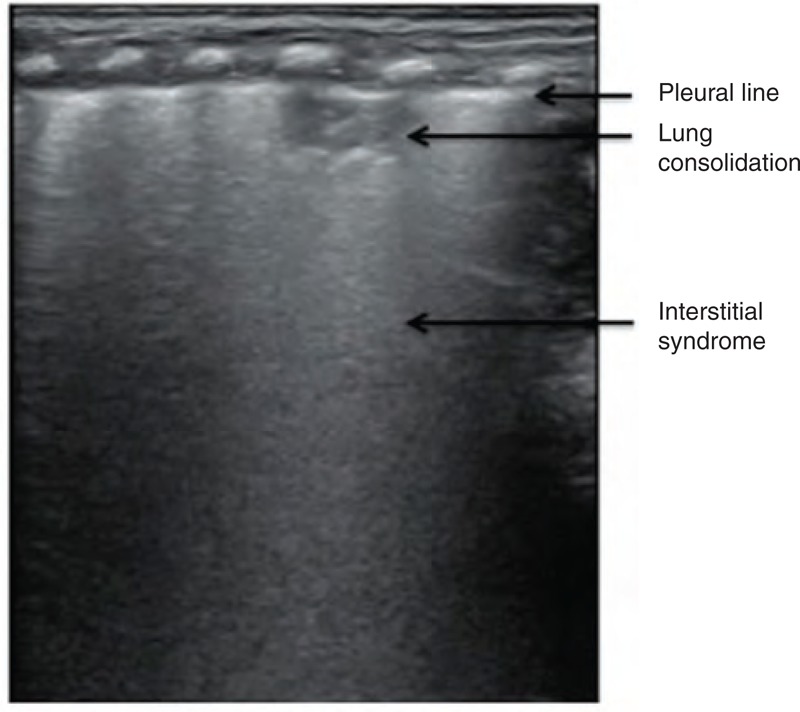
Lung edema with consolidation. An ultrasound showed a small area of focal consolidation beneath the lung pleura (possibly atelectasis or inflammation), whereas the remaining lung field demonstrated interstitial syndrome, suggesting the presence of mild lung edema.

Among the 50 patients enrolled in our study, there were 9 cases of atelectasis (Figure 1) (4 involved BPD coexisting with atelectasis), 4 cases of pneumonia (Figure 2) (2 involved BPD coexisting with pneumonia), 2 cases of severe pulmonary edema (Figure 3) (which did not include mild pulmonary edema experienced during recovery, Figure 4), and 3 cases of pulmonary edema accompanied by focal pulmonary consolidation (Figure 5). These results indicated that more than one-third (18/50 = 36%) of our patients with LTOD either did not actually have BPD or did not have BPD alone. After receiving either endotracheal lavage or pulmonary physical therapy, which resulted in improved atelectasis and aided in the resolution of both inflammation and pulmonary edema, these patients experienced either significant improvements or total resolution of their respiratory symptoms.

## DISCUSSION AND CONCLUSIONS

Lung ultrasound has important value for both the diagnosis and differential diagnosis of lung disease. The diagnosis of lung disease has historically been based on clinical symptoms and laboratory and chest x-ray results, and ultrasound has typically not been utilized in the diagnostic work-up of neonatal lung disease. However, ultrasound has recently been extensively used and is successful in the diagnosis of many types of lung disease.^5–13^ Ultrasound has also become an important tool for patient examination and patient monitoring, as well as for disease management, and is gradually being accepted by clinicians.^14,15^ Because of the extremely high accuracy and reliability of ultrasound, the international evidence-based recommendations for point-of-care lung ultrasound were established in 2012 under the auspices of the International Liaison Committee on Lung Ultrasound,^2^ which has played an important role in promoting more extensive use of lung ultrasound.

Obvious deficiencies exist in the current diagnostic criteria for BPD. Currently, the clinical diagnosis of BPD is made primarily on the basis of the length of time for which an infant has been oxygen dependent, and any infant >28 days of life receiving oxygen (oxygen concentration > 21%) would be diagnosed with BPD.^1^ Because this definition focuses only on the length of oxygen dependence and does not take into account specific reasons for said dependence, this approach clearly has significant limitations and may lead to an overdiagnosis and poor understanding of BPD, eventualities that may result in both unreasonable and unnecessary interventions, including interventions with adverse consequences. It was generally thought that BPD occurs as a result of immature or dysplastic lung tissue, particularly in premature infants. The diagnosis of BPD is clearly incorrect if neonatal oxygen dependence occurs as a result of pulmonary infection, atelectasis, cardiogenic pulmonary edema, or other pathological processes, although BPD may coexist with other types of lung disease, such as pneumonia.

The results of this investigation indicate that LTOD was caused by atelectasis, inflammation, severe pulmonary edema, and coexisting consolidation in more than one-third of the patients enrolled in the study. However, their oxygen dependence either resolved or significantly improved following the resolution of these coexisting conditions. Therefore, these children should not have been diagnosed with BPD, and it is unreasonable for the diagnosis of BPD to remain a part of their medical histories.

Atelectasis is one of the most common pulmonary complications of neonatal lung disease. However, it is difficult to diagnose occult lung atelectasis using traditional chest radiography, whereas chest computed tomography (CT) and lung ultrasound make its diagnosis easier.^6^

Occult lung atelectasis is not easily detected by radiographic examination, and chest CT was not utilized; existing atelectasis necessitated the use of long-term oxygen therapy for affected infants and resulted in BPD. This type of BPD may be considered iatrogenic BPD. It is therefore necessary to revise the diagnostic criteria for BPD and continue studying and utilizing bedside lung ultrasound as an additional diagnostic tool, as this modality has not been universally adopted.^15^

Regarding the advantages of lung ultrasound and the limitations of this study, lung ultrasound has been used successfully in the diagnosis of important lung diseases, including respiratory distress syndrome, transient tachypnea, infectious pneumonia, and pulmonary atelectasis of the newborn, and pneumothorax. Indeed, lung ultrasound has many advantages. First, ultrasound does not expose patients to ionizing radiation and may be performed at the bedside, making it particularly useful for neonates, for whom it is difficult to arrange transport to the radiology department. Second, ultrasound is easy to operate and may be repeated several times per day. Ultrasound may also be useful in guiding emergent life-saving therapies and interventions. Third, ultrasound is a low-cost technique and requires only elementary skills. Fourth, lung ultrasound is highly accurate and reliable in diagnosing pulmonary disease. Therefore, ultrasound may replace chest radiographs and become a first-line imaging modality for the diagnosis of lung disease in advanced NICUs.^14^ Although lung ultrasound helps rule out causes of neonatal LTOD, diagnosing BPD with lung ultrasound is difficult; thus, recognition of the signs of BPD on ultrasound requires further study and represents our current direction of research.

The results of this article indicate that the diagnosis of BPD was either unreasonable or incorrect in at least one-third of patients; therefore, it is necessary to modify the diagnostic criteria for BPD as soon as possible. Meanwhile, it is also necessary to increase the use of lung ultrasound in the NICU.
